# The Dichotomy of Tumor Control by Recruited and Resident Tumor-Associated Macrophages

**DOI:** 10.21203/rs.3.rs-6977440/v1

**Published:** 2025-07-04

**Authors:** Claudia Jakubzick, Soubhik Ghosh, Xin Li, Kavita Rawat, Aishwarya Dighal, Stephanie Kalinowski, Fred Knolling, Carol Ringelberg

**Affiliations:** Dartmouth College; Dartmouth College; Dartmouth College; Washington University School of Medicine; Dartmouth College; Dartmouth college; Geisel School of Medicine at Dartmouth; Dartmouth college

## Abstract

Tumor-associated macrophages (TAMs) play dual roles in cancer, either promoting or suppressing tumor progression, complicating therapeutic approaches. TAMs include recruited macrophages (recMacs), derived from circulating monocytes, and tissue-resident interstitial macrophages (IMs). We recently identified a heterogeneous population of chemokine-expressing IMs, including subsets that support tertiary lymphoid structure (TLS) formation during lung inflammation. Here, we show that IMs can be either pro- or anti-tumorigenic, depending on the subset. Using Pf4 Cx3cr1 mice to deplete CD206hi IMs expressing Cxcl13, Cxcl9, and Cxcl10, we demonstrate their essential role in TLS formation, lymphocyte recruitment, and tumor suppression in melanoma and lung adenocarcinoma. In contrast, Ccl2-expressing IMs promote tumor growth by recruiting pro-tumorigenic recMacs. Spatial transcriptomics confirmed the distinct localization and chemokine profiles of these subsets. Finally, CCR5 blockade with the FDA-approved inhibitor Maraviroc during neoantigen vaccination improved tumor control by preventing the migration of immunosuppressive, antigen-presenting recMacs (moDCs). These findings support the development of macrophage-targeted therapies by identifying pro-tumorigenic subsets and recMac trafficking as actionable targets, while preserving macrophage populations that sustain anti-tumor immunity.

## Introduction

Macrophages are essential immune cells that maintain tissue homeostasis and serve as first responders to infection and injury. In the lung, two major tissue-resident macrophage populations have been described: alveolar macrophages (AMs), which reside in the airspaces, and interstitial macrophages (IMs), which occupy the lung interstitium^1, 2, 3, 4, 5, 6, 7, 8^. While AMs are lung-specific, IMs are also found in other organs and are conserved across species. These two populations differ in localization, transcriptional identity, and immune function^2, 9, 10^. In addition to resident macrophages, a separate population of recruited macrophages (recMacs) arises from circulating monocytes that infiltrate tissues in response to inflammation or tumor development^11, 12, 13^. Within the tumor microenvironment (TME), recMacs can adopt either anti- or pro-tumorigenic phenotypes, or differentiate into monocyte-derived dendritic cells (moDCs)^14, 15^, which transport antigens to lymph nodes and influence adaptive immune responses.

In this study, we refer to IMs as self-renewing, tissue-resident macrophages, and recMacs as short-lived cells derived from monocytes (M0-like) that typically do not persist unless replenishing an empty tissue-resident niche^12, 16, 17^. Conventional methods such as flow cytometry fail to distinguish IMs from recMacs due to shared surface markers. In contrast, single-cell RNA sequencing (scRNA-seq) enables transcriptional resolution, and we previously identified gene signatures that differentiate IMs from recMacs in the lung^18^

Macrophage populations, once considered homogeneous, are now recognized as highly heterogeneous. For example, AMs alone comprise at least 14 transcriptionally distinct subsets, which are not resolved by commonly used surface markers^19, 20^. This internal complexity also complicates the identification of IMs and recMacs in the TME, where markers such as CD11b, Trem2, CD206, and CD169 label multiple macrophage types as well as other myeloid cells. CD206, frequently used to identify pro-tumorigenic macrophages, is variably expressed and not exclusive to macrophages^21, 22, 23^. One study demonstrated that CD206 expression alone is not a reliable marker of pro-tumorigenic phenotype^23^, a conclusion further supported by our current findings. Indeed, both AMs and nearly half of IM subsets express high levels of CD206, yet do not transcriptionally align with either M1- or M2-like states. Instead, AMs and IMs exhibit broad transcriptional diversity, with subsets differentially expressing genes involved in chemokine signaling, growth factor production, metabolic programming, and inflammatory responses^6, 9, 18, 19, 20, 24^. These findings highlight the need for comprehensive transcriptional profiling to define macrophage function in vivo, rather than relying on surface markers alone. Although no current approach, including our own, enables selective depletion of a single macrophage subset, several available genetic models permit functional interrogation of specific macrophage populations.

IMs can be further subdivided based on CD206 expression. CD206^hi^ IMs co-express *CD163* and *Folr2* and exhibit variable levels of *Cx3cr1, MhcII*, and *Lyve1*. CD206^lo^ IMs express *Tmem119, Cd11c, Ccr2*, high levels of *Cx3cr1*, and *MhcII*. Most IMs reside in the bronchovascular interstitium, with smaller populations in the alveolar interstitium, airspaces, and visceral pleura^6, 25, 26^. They are anatomically positioned near nerves and blood vessels and are thought to contribute to tissue development and repair^8, 27^. However, studies in IM-deficient mice suggest these cells also play active roles in regulating immune responses.

To investigate the role of IMs in tumor immunity, we used *Pf4 Cx3cr1* mice, which selectively deplete CD206^hi^ IMs and, to a lesser extent, CD206^lo^ IMs. These include chemokine-producing subsets that express *Cxcl13, Cxcl9, Cxcl10*, and *Ccl24*, chemokines that recruit B cells, T cells, and eosinophils. In models of allergic and infectious inflammation, depletion of these IMs impaired tertiary lymphoid structure (TLS) formation and reduced lymphocyte infiltration^18^. Since TLS presence and B cell abundance correlate with improved prognosis in lung cancer, we hypothesized that specific IM subsets promote anti-tumor responses. We propose that IMs influence tumor immunity in a subset-specific manner. IMs expressing Cxcl13, Cxcl9, and Cxcl10 enhance anti-tumor immunity by promoting TLS formation and lymphocyte recruitment^28, 29, 30^, whereas Ccl2-expressing IMs recruit recMacs, which in turn drive tumor progression through secretion of pro-tumorigenic mediators. Thus, IMs can either support or suppress tumor growth depending on the immune cell populations they attract to the TME. Finally, building on our previous work showing that moDCs can act as antigen-presenting cells that migrate to lymph nodes and induce IL-10-dependent regulatory T cells^13, 31^, we investigated whether blocking CCR5-dependent monocyte lymph node trafficking during neoantigen vaccination could enhance tumor control. Together, these findings refine our understanding of TAM heterogeneity, reveal a functional dichotomy in IM subsets defined by chemokine expression, and point to therapeutic strategies that suppress tumor-promoting recMacs while preserving IM populations that support anti-tumor immunity.

## Results

### Recruited macrophages exhibit pro-tumor transcriptional signatures

**Figure 1:**
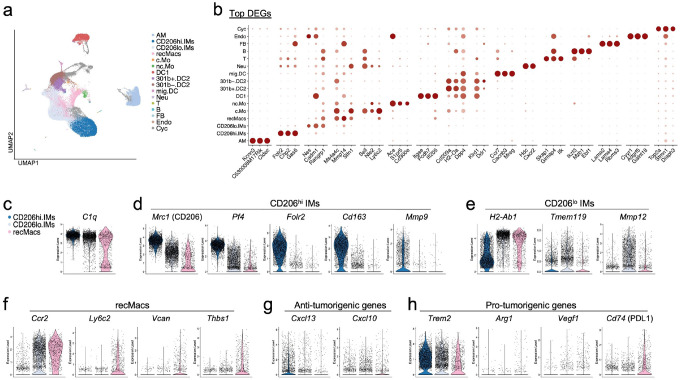
Pulmonary melanoma scRNA-seq identifies pro- and anti-tumorigenic signatures in CD206^hi^ IMs, CD206^lo^ IMs and recMacs (a) UMAP of extravascular immune cells isolated from the lungs of day 16 melanoma burdened C57BL/6 mice, n=3 (b) DEGs defined the unbiased clusters. Alveolar macrophages, AMs; interstitial macrophages, IMs (CD206^hi^ and CD206^lo^, recruited macrophages, recMacs; classical and non-classical monocytes, c.Mo and nc.Mo; dendritic cells, DC1, DC2 (CD301^+^ and CD301^−^); migratory DCs, mig.DCs, neutrophils, Neu; basophils, B; fibroblast,FB; endothelial cells, E; B cells, B; and cycling cells, Cyc. (c-h) Violin plots comparing gene expression in IM subsets and recMacs. (c) Feature plots show the expression of *C1qb* key IM signature genes. (d) Feature plots depict CD206^hi^ IM expression for *Mrc1, Pf4 Folr2, Cd163, and Mmp9*. (*e*) CD206^lo^ IM expression for *H2-Ab1, Tmem119*, and *Mmp12*. (f) Feature plots show recMacs expression for *Ccr2, Ly6c2, Vcan*, and *Thbs1*. (g-h) Feature plots display anti-tumorigenic chemokines *Cxcl13*, and *Cxcl10 by* IMs and pro-tumorigenic genes such as *Trem2* expressed by both recMacs and IMs, and *Spp1, Vegfa, Vcan, Arg1*, and *Cd274* by recMacs.

To determine which macrophage subsets contribute to tumor regression or progression, we isolated extravascular interstitial macrophages (IMs) and recruited macrophages (recMacs) from the lungs of mice bearing pulmonary melanoma (Fig. 1a; Supplementary Fig. 1a–e, second scRNA-seq dataset). Unbiased UMAP clustering identified 16 immune cell populations based on curated and differentially expressed genes (DEGs) (Fig. 1a–b; Supplementary Fig. 1f), after which analysis focused on macrophage subsets. In our previously published scRNA-seq dataset, we defined transcriptional signatures specific to recMacs and IMs under conditions in which circulating monocytes were depleted prior to tissue entry, thereby eliminating recMacs and allowing for clear resolution of IM and recMac clusters^18^. RecMacs selectively expressed *Ly6c2, Vcan, Thbs1*, and higher levels of *Ccr2* and *Fn1*, while IMs expressed elevated levels of *C1q* and *Pf4* (Fig. 1; Supplementary Fig. 1). A third macrophage population consisted of alveolar macrophages (AMs), identified by Ear1 and the top DEG *Cidec*, a marker that appears to be specific to mice^9^. Despite intravascular labeling with anti-CD45 and exclusion during sorting, a small population of intravascular mononuclear phagocytes, including classical and nonclassical monocytes, was still captured in the scRNA-seq dataset (Fig. 1a–b).

While both IMs and some recMacs expressed *C1qb* and *Mrc1* (CD206), CD206^hi^ IMs were further characterized by expression of *Folr2, Cd163, Mmp9*, and *Pf4*, with variable expression of *Lyve1*. In contrast, CD206^lo^ IMs expressed high levels of *Ccr2* and *Mmp12* (Fig. 1c–e; Supplementary Fig. 2). We next examined expression of classical anti- and pro-tumorigenic genes. IMs predominantly expressed anti-tumorigenic including *Cxcl13, Cxcl9*, and *Cxcl10*, whereas both IMs and recMacs expressed the pro-tumorigenic genes such as *Ccl2* and *Trem2*(Fig. 1g; Supplementary Fig. 1g)^32^. Compared to IMs, recMacs were enriched for canonical tumor-promoting transcripts including *Spp1, Vegfa, Arg1*, and *Cd274* (Fig. 1h; Supplementary Fig. 1e) Together, these data suggest that while both populations contribute to immune regulation, recMacs are more transcriptionally aligned with tumor-promoting programs.

### CD206^hi^ IMs limit tumor progression over time by promoting chemokine expression, lymphocyte recruitment, and TLS formation

**Figure 2:**
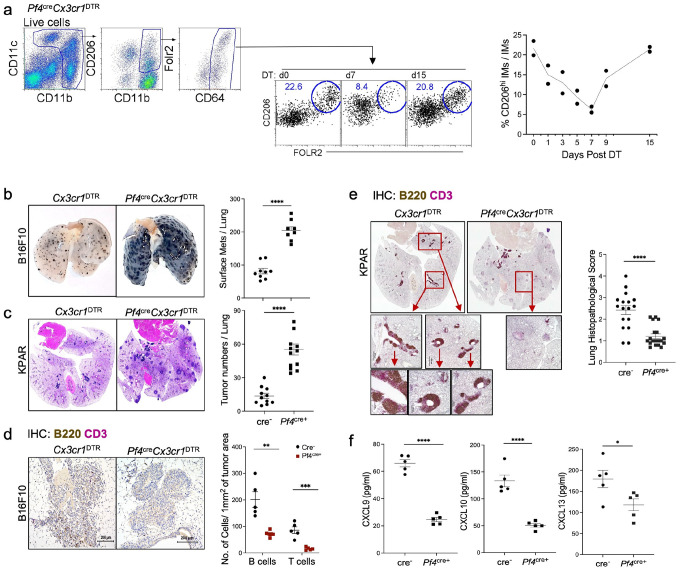
CD206^hi^ IMs promote lymphocyte recruitment and anti-tumor immunity. (a) Gating strategy for CD11b^+^CD64^+^CD206^hi/lo^FOLR2^+/−^ IMs and the time course of CD206^+^FOLR2^+^ IM depletion kinetics in *Pf4*^cre^*Cx3cr1*^DTR^ mice following DT injection on day 0. Three independent experiments were conducted. (b) Representative images of tumor-burdened lungs from cre-*Cx3cr1*^DTR^ and *Pf4*^cre^*Cx3cr7*^DTR^ mice harvested on day 16 after intravenous injection of melanoma cells. Plot shows the number of surface metastases per lung. Four independent experiments were conducted, with *n* = 4–5 per group. (c) Representative hematoxylin and eosin-stained lung sections from cre-*Cx3cr1*^DTR^ and *Pf4*^cre^*Cx3cr1*^DTR^ mice harvested on day 16 after injection of KPAR1.3 adenocarcinoma cells. Plot quantifies the number of tumors per section. Four independent experiments were conducted, with *n* = 5 per group. (d) Representative IHC sections stained for B220 (brown) and CD3e (purple). The plots show the number of T cells and B cells infiltrating the TME in cre-*Cx3cr1*^DTR^ and *Pf4*^cre^*Cx3cr1*^DTR^ mice. Three independent experiments were conducted, with *n* = 5 per group. (e) Representative IHC sections stained for B220 (brown) and CD3e (purple), showing prominent tertiary lymphoid structures (TLS) in cre-*Cx3cr1*^DJR^ lungs, whereas *Pf4*^cre^*Cx3cr1*^DTR^ lungs exhibited virtually no TLS. Whole lung images and magnified views of TLS are presented. The plots show lung histopathology scores in *cre-Cx3crlDTR* and *Pf4*^cre^*Cx3cr1*^DTR^ mice. Three independent experiments were conducted, with *n* = 5 per group. (f) ELISA analysis of CXCL9, CXCL10, and CXCL13 levels in homogenized lungs from cre-*Cx3cr1*^DTR^ and *Pf4*^cre^*Cx3cr1*^DTR^ mice on day 16 after KPAR1.3 adenocarcinoma injection. Two independent experiments were conducted, with *n* = 5 biologically independent samples per group. p-values were calculated using a two-sided Student’s t-test. *p < 0.05; ** *p* < 0.01; **** *p* < 0.001

IMs differentiate into at least ten distinct chemokine-expressing subsets, several of which play protective roles in pulmonary inflammation and infection^18^. To assess their function in cancer, we used *Pf4*^*cre*^*Cx3cr1*^*DTR*^ mice, which predominantly deplete **CD206**^**hi**^ IMs, including those that express *Cxcl13* (for B cell chemotaxis), *Cxcl9* and *Cxcl10* (for NK and T cell recruitment), as well as *Ccl6, Ccl8, Ccl9*, and *Ccl24*^18^. While Pf4 is also expressed in megakaryocytes and peritoneal macrophages, these cells do not express *Cx3cr1* and thus remain intact in this model. *F0LR2*^+^*CD206*^*hi*^ IMs are maximally depleted by day 7 following diphtheria toxin (DT) injection, with full recovery by day 15 (Fig. 2a)^18^.

To examine the role of **CD206**^**hi**^ IMs in tumor control, we intravenously injected melanoma (B16F10) and lung adenocarcinoma (KPAR1.3) cells into *Pf4*^*cre*^*Cx3cr1*^*DTR*^ and *Cx3cr1*^*DTR*^ littermate control mice. DT was administered on days 3 and 7 to allow equivalent tumor seeding prior to IM depletion, and tumor burden was assessed on day 16 (Fig. 2b–c; Supplementary Fig. 2a–b). Across both models, mice lacking **CD206**^**hi**^ IMs exhibited significantly increased tumor burden relative to DT-treated controls. A single DT dose at day 4 was also sufficient to produce a similar phenotype (Supplementary Fig. 2c), indicating that early loss of IMs permits unchecked tumor growth.

We next assessed lymphocyte infiltration and TLS formation in tumor-bearing lungs using immunohistochemistry. Although the melanoma model does not typically form TLS, B and T cells were readily observed in the TME and peribronchial regions of control mice (Fig. 1d; Supplementary Fig. 2d). In contrast, **CD206**^**hi**^ IM-depleted mice exhibited a near-complete loss of lymphocyte infiltration (Fig. 2d). In the lung adenocarcinoma model, which supports TLS formation^33^, **CD206**^**hi**^ IM depletion led to significantly greater tumor burden accompanied by a striking absence of TLS compared to IM-sufficient controls (Fig. 2e). To determine whether IM depletion impacted local chemokine expression, we measured cytokine levels in tumor-bearing lungs. IM-deficient mice exhibited markedly reduced levels of CXCL9, CXCL10, and CXCL13 (Fig. 2f). Together, these findings demonstrate that **CD206**^**hi**^ IMs are critical for orchestrating chemokine production, lymphocyte recruitment, and TLS formation, and that their loss promotes tumor outgrowth.

### Spatial mapping reveals compartmentalized chemokine expression by recMacs and IMs in the TME

**Figure 3:**
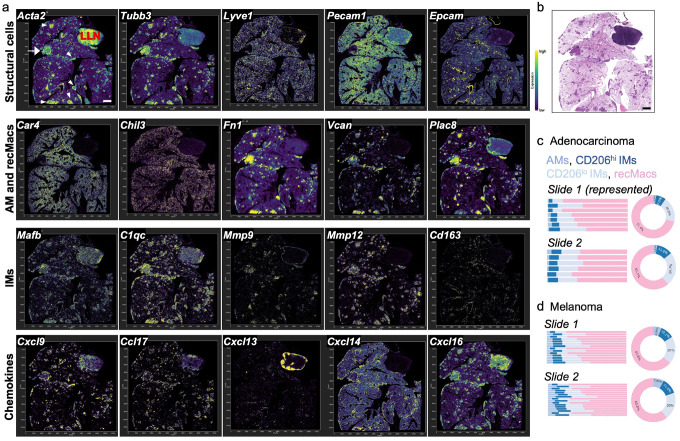
Spatial transcriptomics of adenocarcinoma reveals distinct chemokine patterns and a high abundance of recMac and CD206^lo^ IMs compared to CD206^hi^ IMs, with virtually no AMs in the TME. (a) Spatial expression analysis of d16 KPAR1.3 adenocarcinoma lungs shows high concentration of Actin (*Acta2*), aiding in TME identification. White arrows indicate tumor nodules. A large white arrow, to the left of *Acta2* image, pointing at a large tumor, highlights structural cells within the TME across the top row. *In situ* gene expression reveals distinct cell types, including innervated nerves (*Tubb3*), lymphatic vessels (*Lyve1*), blood vessels (*Pecam1*), and epithelial cells (*Epcam*). Markers for macrophage populations include alveolar macrophages (*Chil3, Car4*), recMacs (*Fn1, Vcan, Plac8*), IMs (*Mafb, C1qc*), CD206^hi^ IMs (*CD163, Mmp9*), and CD206^lo^ IMs and recMacs (*Mmp12*). Additionally, chemokines (*Cxcl9, Ccl17, Cxcl13, Cxcl14, Cxcl16*) are represented. A control slide without tumors is shown in Supplementary Fig 4. (b) Hematoxylin and eosin-stained section of a spatial transcriptomic adenocarcinoma lung. (c-d) Spatial transcriptomics and quantification of extravascular macrophages within the tumor TME in adenocarcinoma and melanoma. All spatial samples are shown; n = 2 per tumor type.

We performed spatial transcriptomics on four tumor-bearing lungs, two with melanoma and two with adenocarcinoma, using the 10x Xenium platform and a predefined gene panel enriched for myeloid markers, chemokines, and stromal components (Supplementary Fig. 3). This in situ hybridization approach enabled subcellular localization of recMacs, IMs, AMs, chemokine expression, and associated immune populations within the TME. Representative data from the four samples are shown (Fig. 3a). One section included a large lung-draining lymph node, which served as an internal quality control for the Xenium platform and chemokine expression. As expected, *Cxcl13* localized to the B cell zone, while *Cxcl16, Ccl17*, and *Ccl22*, typically expressed by dendritic cells, were enriched in the T cell zone (Fig. 3a; Supplementary Fig. 4), validating the spatial specificity of our dataset.

Tumor nodules were readily identifiable by *Acta2* expression and H&E morphology, with non-hematopoietic components outlining the lung architecture (Fig. 3a–b, white arrows). The TME was highly innervated, as shown by *Tubb3* and *Nes* expression, whereas lymphatic (*Lyve1, Pdpn*), vascular (*Pecam*), and epithelial (*Epcam*) markers were relatively sparse (Fig. 3a; Supplementary Fig. 4). *Epcam* clearly outlined the bronchial airway epithelium (Fig. 3a). Graph-based clustering and DEG analyses from the 10x Xenium pipeline were used to statistically define cell types based on known marker combinations, enabling identification and quantification of AM, IMs and recMacs (Supplementary Fig. 3). Spatial localization of these macrophage subsets was then validated using the selection tool in Xenium Explorer 3 to retrieve and map relevant cell IDs in the TME (Fig. 3c–d). Spatial analysis of myeloid populations revealed that AMs (*Car4, Chil3, Ear1*) were largely excluded from the TME, whereas dendritic cells (*Zbtb46, Flt3, Xcr1*) were distributed throughout (Fig. 3a; Supplementary Fig. 4). RecMacs, marked by *Fn1, Vcan1, Plac8, Clec4n, Cd9*, were abundant in the TME^18^, as were IMs, marked by *Mafb, C1q, Mmp9*, and *Mmp12* (Fig. 3a; Supplementary Figs. 3–5). **CD206**^**hi**^ IMs (*Folr2, Cd163, Mmp9*) were primarily localized to the bronchial airways and visceral pleura, with a small subset of Mmp9-expressing cells sparsely distributed in the TME. In contrast, CD206^lo^ IMs (*Mmp12*) and recMacs were highly enriched in tumor-dense regions (Fig. 3a; Supplementary Figs. 3–5).

We next examined spatial patterns of chemokine expression across the TME. Multiple chemokines including *Ccl3, Ccl4, Ccl6, Ccl7, Ccl8, Ccl9, Ccl17, Ccl22, Cxcl9, Cxcl10, Cxcl13, Cxcl3, Cxcl14*, and *Cxcl16* were detected within the tumors, with subset- and region-specific expression (Fig. 3; Supplementary Fig. 3). Notably, *Cxcl13* is also highly present along the bronchial airways where *Cd163*^+^ *Folr2*^*+*^ CD206^hi^ IMs are located and TLS forms. *Cxcl14*, a CXCR4-inhibitory ligand, localized to the outer tumor margins, whereas *Cxcl16*, a CXCR6 ligand important for effector T cells and ILC2s, was enriched in the tumor core (Fig. 3a). Overall, these spatial transcriptomic data reveal that AMs, and to some extent **CD206**^**hi**^ IMs, are largely excluded from heavily tumor-infiltrated areas, while recMacs and **CD206**^**l**o^ IMs populate the TME and contribute to its chemokine landscape.

### IM-derived CCL2 promotes tumor growth by recruiting recMacs

**Figure 4:**
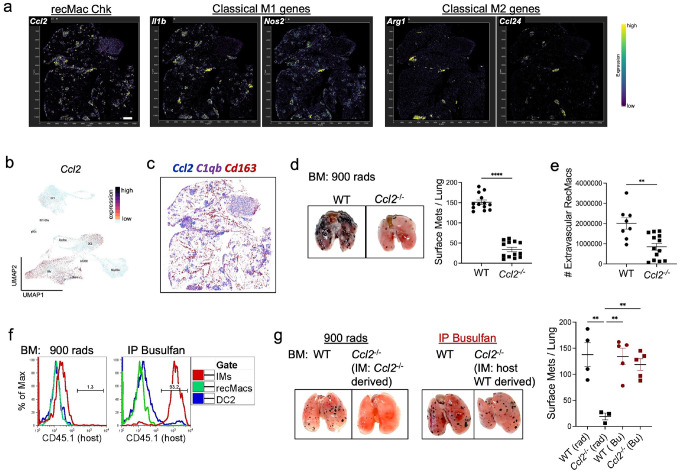
*Ccl2* expression by IMs is critical for recMac recruitment. (a) Spatial expression analysis of d16 KPAR1.3 adenocarcinoma lungs reveals high *Ccl2* expression in the TME, along with classical M1 pro-inflammatory genes *Il1b* and *Nos2*, as well as M2 reparative genes *Arg1* and *Ccl24*. (b) UMAP visualization of *Ccl2* expression in myeloid cells, illustrated with labeled second dataset, Supplementary Fig. 1. (c) Spatial transcriptomics overlay of three genes *Ccl2, C1qb* and *Cd163*. (d) Irradiated (900 rad) CD45.1 mouse recipients were reconstituted with CD45.2 WT and *Ccl2*^−/−^ bone marrow. The representative image shows mice on day 16 post-melanoma cell injection. To the right, quantification of the number of surface metastases and (e) number of recMacs per lung. Three independent experiments were conducted, with *n* = 4–5 per group. (f) Four weeks after busulfan injection, myeloid cells were examined: host CD45.1 IMs were preserved, while monocytes and DCs were donor derived (CD45.2). Three independent experiments were conducted, with *n* = 3–4 per group. (g) Four cohorts of mice (Irradiated, IMs are WT-derived and *CclZ*^−/−^ derived; Busulfan-treated: IMs are WT-derived and WT-derived) were injected with B16F10 melanoma cells and examined for tumor growth. Scatter plot quantifies the number of surface metastases per lung. Two independent experiments were conducted, with *n* = 3–4 per group. *p*-values were calculated using a two-sided Student’s t-test. ** *p* < 0.01; **** *p* < 0.001

While IMs facilitate lymphocyte recruitment, they can also drive tumor progression by recruiting reparative type 2 immune cells such as ILC2s and IL-4–producing eosinophils^34^. In addition to classic M1 and M2 gene signatures, we observed strong expression of *Ccl2*, a key chemokine for angiogenesis and monocyte recruitment, across CD206^hi^ and CD206^lo^ IMs, recMacs, and conventional dendritic cells (DCs) in tumor-bearing lungs (Fig. 4a–b)^14, 35^. Spatial transcriptomics showed that *Ccl2* expression was concentrated within tumor regions (Fig. 4c). To determine whether IM-derived *Ccl2* specifically drives tumor progression, we first created bone marrow (BM) chimeras by lethally irradiating CD45.1 wild-type hosts and reconstituting them with either CD45.2 WT or *Ccl2*^−/−^ BM, thereby preserving non-hematopoietic CCL2 (e.g., from endothelial cells)^36^. The results mirrored those observed in global *Ccl2*^−/−^ mice, hematopoietic loss of CCL2 significantly reduced tumor burden and decreased extravascular recMac accumulation compared to WT chimeras (Fig. 4d–e; Supplementary Fig. 5).

However, full-body irradiation depletes all hematopoietic sources of CCL2, not just IMs. To selectively interrogate the role of IM-derived CCL2, we used busulfan, a myeloablative agent that spares long-lived tissue-resident IMs while replacing circulating monocytes, recMacs, and DCs with donor-derived cells. Four weeks post-treatment, host-derived IMs persisted, whereas donor-derived cells populated the peripheral myeloid compartment (Fig. 4f). In this setting, only busulfan-treated mice retained *Ccl2*-expressing IMs. Upon tumor challenge, only irradiated mice lacking Ccl2-expressing IMs showed reduced tumor burden, while busulfan-treated mice with preserved Ccl2-expressing IMs developed larger tumors (Fig. 4g; Supplementary Fig. 6). These findings suggest that IM-derived CCL2, rather than CCL2 from endothelial cells or recMacs, is essential for recMac recruitment and tumor progression.

### RecMacs act as immunosuppressive antigen-presenting cells during cancer vaccination

In addition to their local roles in the TME, recMacs can function as antigen-presenting cells in the lymph node, where they dampen adaptive immune responses. We have previously shown that moDCs induce regulatory T cells and suppress both type 2 allergic inflammation and cytotoxic T cell responses to tumor neoantigens^13, 31^. However, prior studies did not assess the impact of transient CCR5 inhibition, a clinically relevant strategy.

**Figure 5:**
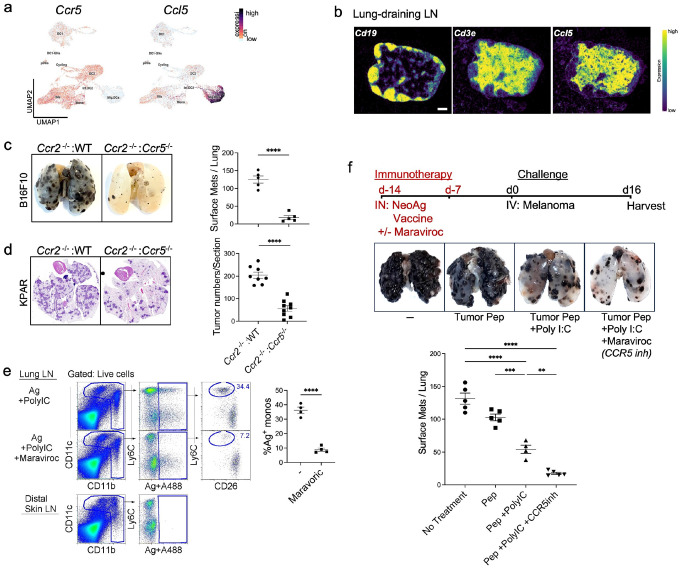
Inhibiting lymph node antigen-bearing moDC with Maraviroc during a prophylactic neoantigen vaccine enhances anti-tumor immunity. (a) UMAP visualization of *Ccl5* and *Ccr5* expression in myeloid cells. (b) Spatial expression of *Cd19, Cd3e*, and *Ccl5* in a lung-draining lymph node. (c) Representative images of tumor-burdened lungs from *Ccr2*^−/−^:WT and *Ccr2*^−/−^:*Ccr5*^−/−^ reconstituted mice harvested on day 16 post-melanoma cell injection. Plot showing the number of surface metastases per lung. Two independent experiments were conducted with *n* = 5 for each group. (d) Representative hematoxylin and eosin-stained sections of lungs from bone marrow chimeric *Ccr2*^−/−^:*WT.1* and *Ccr2*^−/−^*:Ccr5*^−/−^ mice harvested on day 16 post KPAR1.3 adenocarcinoma cell injection. The plot quantifies the number of tumors per section. Two independent experiments with *n* = 5 for each group are represented. (e) WT mice were treated i.p. with or without 300 μg Maraviroc (CCR5 inhibitor) 4 h prior to i.n. delivery with 5 μg OVA-Alexa488 and 50 μg Poly I:C. 24 h post antigen delivery lymph nodes were harvested. Scatter plot left to right displays gated myeloid cells, then gated antigen-bearing cells, and then antigen-bearing Ly6C^+^ moDCs (circled) and CD26^+^ DCs. Three independent experiments were conducted with *n* = 4–5 for each group. (f) Representative image of lungs from prophylactically treated WT mice injected with tumor peptides alone, tumor peptides + Poly I:C, and tumor peptides + Poly I:C + Maraviroc (*Ccr5* inhibitor) 14 and 7 days prior to tumor challenge. The plot shows the number of surface metastases per lung. Two independent experiments were conducted with *n* = 5 for each group. *p*-values were calculated using a two-sided Student’s *t*-test. ** *p* < 0.01; **** *p* < 0.001

We also previously demonstrated that moDC migration from the periphery to draining lymph nodes requires CCR5 expression by monocytes, since they lack CCR7, and CCL5 production by mature DCs^13^ To selectively impair monocyte migration, without affecting DC migration, we generated BM chimeras using a mixture of 80% *Ccr2*^−/−^ and 20% Ccr5^−/−^ or wild-type BM cells. This strategy yields circulating monocytes that lack CCR5, while preserving CCR5-dependent functions in other hematopoietic lineages. Because DCs do not rely on CCR2 or CCR5 for lymph node entry or antigen presentation^31^, this approach allowed us to isolate the role of monocyte-derived antigen-presenting cells. As anticipated, mice with CCR5-deficient monocytes exhibited significantly reduced tumor burden compared to WT controls (Fig. 5c–d), consistent with a role for CCR5^+^ monocytes in suppressing anti-tumor immunity.

To directly assess the immunosuppressive role of CCR5^+^ monocytes during the priming phase of neoantigen vaccination, we used a prophylactic vaccination strategy. First, we confirmed that Maraviroc, a CCR5 inhibitor, selectively blocks the migration of antigen-bearing monocytes, but not dendritic cells, to the draining lymph node. Wild-type mice were intranasally administered fluorescently labeled neoantigen plus Poly I:C. Twenty-four hours later, antigen-loaded Ly6C^+^ monocytes and CD26^+^ DCs were observed in the lung-draining lymph node (Fig. 5e)^13^. Mice pre-treated with Maraviroc four hours prior to vaccination showed a marked reduction in antigen-bearing monocytes, while dendritic cell migration remained unaffected (Fig. 5e)^13^. Given the short half-life of Maraviroc (~16 hours), CCR5 inhibition was limited to the vaccination period, with no effect during the tumor challenge. Mice receiving both neoantigen vaccine and Maraviroc exhibited significantly greater anti-tumor protection compared to those receiving vaccine alone, neoantigen alone, or no treatment (Fig. 5f; Supplementary Fig. 7). These findings reveal that recMacs suppress immunity both within the TME and by presenting antigen in the lymph node, and suggest that transient blockade of monocyte migration enhances neoantigen vaccine efficacy.

## Discussion

Macrophages are highly plastic cells that can either suppress or promote tumor progression depending on their ontogeny, spatial context, and transcriptional state. In this study, we dissected the functional dichotomy between two major macrophage populations in lung cancer: recMacs and long-lived, self-renewing IMs. Leveraging transcriptional, spatial, and macrophage depletion approaches, we demonstrate that a subset IMs and recMacs exert opposing roles in tumor immunity, with distinct temporal and spatial dynamics.

Our data show that **CD206**^**hi**^ IMs, a subset enriched along the bronchial airways and pleura, are critical for lymphocyte recruitment. These IMs produce key chemokines, including *Cxcl13, Cxcl9*, and *Cxcl10*, which support the recruitment and spatial organization of T and B cells within the TME and formation of TLS along the bronchial airways^18^. Spatial transcriptomic analysis confirmed the localization of chemokine-expressing IMs near and within the TME and revealed the inclusion of recMacs and *Ccl2*-expressing IMs in tumor-dense regions and the exclusion of CD206^hi^ IMs, suggesting that tumor architecture imposes spatial constraints on anti-tumoral IM localization and function. Depletion of **CD206**^**hi**^ IMs impaired TLS formation, reduced chemokine levels, and promoted tumor growth, underscoring their essential role in orchestrating local anti-tumor immunity.

In contrast, recMacs, which infiltrate the tumor core, are enriched for pro-tumorigenic programs. These cells express angiogenic factors *(Vegfa, Spp1*), immune checkpoint molecules (*Cd274*), and the chemokine *Ccl2*, which promotes further monocyte recruitment. Using spatial transcriptomics and scRNA-seq, we found that *Ccl2* was primarily expressed within the TME by recMacs and a subset of *Ccl2*-expressing IMs. Functional studies using BM chimeras revealed that hematopoietic deletion of *Ccl2* significantly reduced both tumor burden and the presence of extravascular recMacs, demonstrating that macrophage-intrinsic CCL2 promotes tumor progression. Since *Ccl2* is expressed by both CD206 IM subsets, it is possible that each contributes to tumorigenesis.

We also identify an underappreciated immunosuppressive role for recMacs in the draining lymph node. Building on prior work showing that moDCs induce regulatory T cells in allergic and cancer settings^13, 31^, we show that transient inhibition of CCR5, required for monocyte lymph node migration, enhances the efficacy of neoantigen-based cancer vaccination. Maraviroc, a clinically approved CCR5 antagonist, selectively blocked antigen-bearing monocytes from reaching the lymph node without affecting DC migration, and in prophylactic settings, markedly improved anti-tumor responses. These findings implicate lymph node-trafficking moDCs as critical immunoregulatory agents and suggest that targeting monocyte migration may synergize with immunotherapeutic strategies.

Accurately defining macrophage function in vivo requires analyzing multiple transcriptional genes rather than relying solely on surface marker expression. A critical strength of this study is its translational relevance. Prior work has shown strong transcriptional and functional conservation of myeloid populations, particularly monocytes and interstitial macrophages, between mice and humans^9, 18, 37^. Thus, our findings offer immediate insight into human tumor biology. Together, our integrated approach combining spatial transcriptomics with functional depletion and vaccination models provides a framework for dissecting the spatial and functional complexity of myeloid cells in other tissue settings.

## Methods

### Mice

C57BL/6 Ly5.1 (CD45.1) and Ly5.2 (CD45.2) WT mice were purchased from Charles River/NCI. *Pf4*^*Cre*^ (*C57BL/6-Tg (Pf4-icre) Q3Rsko/J*)*, Cx3cr1*^*DJR*^ (*B6N. 129P2-Cx3cr1tm3(Hbegf)Litt/J*)*, Cc2*^−/−^ *{86.129S4-Ccl2tm1Rol/J*)*, Ccr5*^−/−^ (*B6.129P2-Ccr5tm1Kuz/J*), and *Ccr2*^−/−^ (*B6.129S4-Ccr2tm1Ifc/J*) mice were from Jackson Labs. All mice were bred in-house, genotyped before studies, and used at 6–12 weeks of age. Experiments were performed on age-matched cohorts. *Pf4*^*Cre*^*Cx3crl*^DTR^ mice were compared to *Cre-Cx3cr1*^DTR^ littermate controls in BM chimera studies. Mice were housed under specific-pathogen-free conditions at Dartmouth Hitchcock Medical Center.

#### Ethics statement.

All procedures followed protocol #00002229 approved by the Dartmouth College Institutional Animal Care and Use Committee.

### Bone marrow chimeras

Six-week-old Ly5.1 (CD45.1) WT mice underwent lethal irradiation with a single 900 rad dose. 25 mg/kg of busulfan was used, instead of radiation, to retain host IMs. Following irradiation, mice received 5×10^6^ donor BM cells intravenously from the following genotypes: *Ccr2*^−/−^: WT (80:20 ratio), *Ccr2*^−/−^; *Ccr5*^−/−^ (80:20 ratio), or pure BM chimeras from *Pf4*^cre^*Cx3cr1*^DTR^, Cre-*Cx3cr1*^DTR^, *Ccl2*^−/−^, or WT donors. Chimerism was verified using congenic markers before experimental use.

Melanoma (B16F10) and were cultured according to ATCC and the adenocarcinoma (KPAR1.3) cell line was kindly provided by Dr. Julian Downward^33^. *Pf4*^cre^*Cx3cr1*^DTR^ were given 700ng iv diptheria toxin (DT) at day 0 for time course; and days 3 and 7 (or single dose at day 4) for cancer models. Cells were maintained in DMEM (ATCC 30–2002) supplemented with 10% heat-inactivated fetal bovine serum, 1% L-glutamine, and 1% Penicillin-Streptomycin. Cells were harvested using Trypsin-EDTA, washed with PBS and HBSS, and 4X10^5^ cells were injected intravenously into mice via the tail vein. On day 16, lungs were perfused with PBS, inflated with 0.5% agarose, and fixed overnight in 10% neutral-buffered formalin (NBF) at 4°C. Tumor metastases were counted the following day. For the B16F10 model, tumors were categorized into four size groups and counted across all lobes using a blinded approach. In the KPAR1.3 model, tumors were quantified with hematoxylin and eosin (H&E)-stained sections and plotted as tumor number per section.

#### ELISA chemokine protein analysis:

Mouse IP-10 (CXCL10), CXCL13, and CXCL9 levels were quantified using Invitrogen ELISA kits following the manufacturer’s instructions.

### Flow cytometry

Lungs were perfused with PBS, minced, and digested in 1 ml solution of 2.5 mg/ml collagenase D and 400 μg/mL Liberase TM in RPMI at 37°C for 30 minutes. Digestion was stopped with 100 μl of 100mM EDTA. The cell suspensions were filtered through a 70 μm filter and centrifuged at 300 g for 5 minutes. Samples were stained with monoclonal antibodies (mAbs) and isotype controls from BioLegend or ebioscience, including Alexa488-conjugated CD206, CD4, and CD26; PE-conjugated CD206, CD45.1, and CD26; PerCP-Cy5.5–conjugated CD64, XCR1, Ly6C, and CD8; PE-Cy7–conjugated CD11c; BUV395-conjugated CD11b; APC-conjugated CD88, FOLR2, and CD19; APC-Cy7–conjugated Ly6C and CD45; BV421-conjugated Ly6G and SiglecF; and BV510-conjugated MHCII and CD45.2. The viability dye DAPI was added immediately before sample acquisition on a BD Symphony A3 analyzer. Data were analyzed using FlowJo software. For extravascular leukocyte analysis, mice were injected intravenously with 5 μL anti-CD45 in 200 μL PBS five minutes before sacrifice to exclude intravascular cells.

### CCR5 inhibitor study

WT mice were injected intraperitoneally with 300 μg of Maraviroc (Cayman #14641). Four hours later, for migration studies, mice received intranasally 5 μg of OVA-Alexa-488 and 50 μg Poly:IC 50 μg Poly:IC, after 24hr mice were harvested for the analysis of antigen-bearing cell migration. For immunotherapy, mice received an intranasal immunization with 50 μL containing 20 μg Pmel17, 20 μg Trp2, and 50 μg Poly:IC. The same immunization was repeated on day 7. On day 14, mice were injected intravenously with 1X10^6^ B16F10 cells. On day 30, lungs were harvested for tumor quantification.

### Microscopy

Lungs were perfused with PBS, inflated with 10% neutral-buffered formalin, and paraffin-embedded. Sections (5 μm) were stained with H&E and imaged at x200 magnification using a Keyence BZ-X800 microscope. For histopathological scoring, infiltrates were assessed based on severity, with a scale from 0 (no infiltrates) to 4 (severe infiltrates with complete collars thicker than 10 cells). Each lobe was scored separately, and the average histopathology score was reported. *Immunohistochemistry*. 4um sections were stained with Rat anti-mouse/human B220 (BioLegend #103226) and Rabbit anti-mouse CD3e (Cell Signaling #99940).

### Xenium sample preparation and data acquisition

#### Sample Processing:

*Two mice with* B16 tumor-bearing C57BL/6 mice and *two* mice with KPAR1.3 tumors. For 10X Genomics Xenium spatial transcriptomics, mice were perfused with 10% Neutral Buffered Formalin (NBF) to remove circulating blood. The lungs were then inflated with NBF to preserve tissue architecture and fixed by submersion in 10% NBF for 12 hours at room temperature. After fixation, lung tissues were processed for paraffin embedding, and formalin-fixed paraffin-embedded (FFPE) blocks were prepared. Sections were then cut at 5um thickness onto Xenium slides in the Pathology Shared Resource at Dartmouth (RRID: SCR_023479) according to 10x Genomics protocol CG000580). Slides were then transferred to the Genomics and Molecular Biology Shared Resource (RRID:SCR_021293) and processed following the manufacturer’s instructions for FFPE tissue sections (Protocol: CG000581) followed by probe hybridization, ligation and amplification (Protocol: CG000582). Slides were run on a Xenium Analyzer instrument running Xenium instrument software version 2.0.1.0 and On-Board Analysis software version 2.0.0.10 to produce the output data bundle used for downstream analysis. Following the Xenium run, slides were H&E stained on a Sakura Tissue-Tek Prism stainer and whole slide imaging conducted at 40x magnification using an Aperio GT450 instrument (Leica). Xenium spatial transcriptomics analysis was then processed and performed using Xenium Explorer 3 (10x Genomics), with cell population identification conducted using R v.4.2.

#### Data Preparation

Graph-based clustering results and differentially expressed genes (DEGs) from the 10x Xenium pipeline were utilized for cell-type identification based on known marker combinations, enabling the identification of IM clusters. Spatial localization of IM subsets was explored using the selection tool in Xenium Explorer 3, retrieving relevant cell IDs. Additional analyses were conducted in R v.4.2 using the Seurat Xenium pipeline.

### Single-cell RNA sequencing data and references

Single-cell RNA sequencing data used in this study were obtained from mouse pulmonary cells post-B16F10 exposure n=3 B16 samples were used and pulled together (Fig. 1). Second set (Supplementary Fig. 1 data) n=6 B16 samples were used and pulled together, GSE22566. To differentiate intravascular and extravascular leukocytes, mice were injected intravenously with APC-Cy7-conjugated anti-CD45 antibody 5 minutes before harvest. Lung single cell suspensions were sorted to enrich for extravascular monocyte-macrophage populations, CD64^+^CD11b^+^ cells, using the FACS Aria Fusion (BD Biosciences). Approximately 30,000 cells per sample were loaded on the Chromium Next GEM Single Cell 3′ Platform (10x Genomics) and sequenced on an Illumina NextSeq 500/550 with an average depth of approximately 50,000 reads per cell.

#### Data preparation

Raw sequencing reads were demultiplexed and mapped to the GRCm38 genome using CellRanger v6.1. Data processing and analysis were performed in R v4.2 and Python v3.6, with Seurat v4.3 used for data integration and visualization. Cell type identification followed methods detailed in^18^, wherein IMs and recMacs were distinguished based on characteristic marker genes and clustering profiles. The processed data are available in the Gene Expression Omnibus under accession codes GSE225664 and GSE225667 and are accessible for online visualization at UCSC Cell Browser.

### Statistics and reproducibility

All measurements were taken from distinct samples and the number of individuals in each experiment or analysis is clearly indicated either in the text or in Fig legends. Significance was evaluated using a two-tailed Student’s *t*-test. Data distribution for the transgenic mouse experiment was assumed to be normal but this was not formally tested. In the selection of experimental cohorts of mice, randomization was not the dominant driver of the process. Littermate controls were assigned appropriately to match mice that were genetically altered, so that controls were tested side by side with those bearing a different genotype. Experimental analysis was carried out so that for any given length of a protocol, all experimental cohorts were dealt with simultaneously; no one whole group was processed first before the next, but the cohorts were evenly distributed throughout the procedure. All samples were given a code name and this was processed without reference to its cohort features until the end of the experiment. Data collection and analysis were performed blind to the genotypes of the mice. The investigators were blinded to allocations during experiments and outcome assessment. No animals or data points were excluded from the study.

## Supplementary Files

This is a list of supplementary files associated with this preprint. Click to download.

- SupplementaryFilesfinal.docx
